# Regional mapping of myocardial hibernation phenotype in idiopathic end-stage dilated cardiomyopathy

**DOI:** 10.1111/jcmm.12198

**Published:** 2014-01-20

**Authors:** Vincenzo Lionetti, Marco Matteucci, Marco Ribezzo, Dario Di Silvestre, Francesca Brambilla, Silvia Agostini, Pierluigi Mauri, Luigi Padeletti, Alessandro Pingitore, Luisa Delsedime, Mauro Rinaldi, Fabio A Recchia, Angela Pucci

**Affiliations:** a Laboratory of Medical Science, Institute of Life Sciences, Scuola Superiore Sant'AnnaPisa, Italy; b Fondazione CNR-Regione Toscana “G. Monasterio”Pisa, Italy; c Cardiac Surgery Department, San Giovanni Battista University HospitalTurin, Italy; d Institute for Biomedical Technologies (ITB)-CNRSegrate, Milan, Italy; e Department of Medical and Surgical Critical Care, University of FlorenceFlorence, Italy; f Institute of Clinical Physiology-CNRPisa, Italy; g Histopathology Department, S. Giovanni Battista University HospitalTurin, Italy; h Department of Physiology, Temple University School of MedicinePhiladelphia, PA, USA; i Department of Pathology, University Hospital PisaPisa, Italy

**Keywords:** pathologic features, hibernating myocardium, chronic heart failure, idiopathic dilated cardiomyopathy, ischaemic microenvironment, nestin

## Abstract

Myocardial hibernation (MH) is a well-known feature of human ischaemic cardiomyopathy (ICM), whereas its presence in human idiopathic dilated cardiomyopathy (DCM) is still controversial. We investigated the histological and molecular features of MH in left ventricle (LV) regions of failing DCM or ICM hearts. We examined failing hearts from DCM (*n* = 11; 41.9 ± 5.45 years; left ventricle-ejection fraction (LV-EF), 18 ± 3.16%) and ICM patients (*n* = 12; 58.08 ± 1.7 years; LVEF, 21.5 ± 6.08%) undergoing cardiac transplantation, and normal donor hearts (N, *n* = 8). LV inter-ventricular septum (IVS) and antero-lateral free wall (FW) were transmurally (*i.e*. sub-epicardial, mesocardial and sub-endocardial layers) analysed. LV glycogen content was shown to be increased in both DCM and ICM as compared with N hearts (*P* < 0.001), with a U-shaped transmural distribution (lower values in mesocardium). Capillary density was homogenously reduced in both DCM and ICM as compared with N (*P* < 0.05 *versus* N), with a lower decrease independent of the extent of fibrosis in sub-endocardial and sub-epicardial layers of DCM as compared with ICM. HIF1-α and nestin, recognized ischaemic molecular hallmarks, were similarly expressed in DCM-LV and ICM-LV myocardium. The proteomic profile was overlapping by ˜50% in DCM and ICM groups. Morphological and molecular features of MH were detected in end-stage ICM as well as in end-stage DCM LV, despite epicardial coronary artery patency and lower fibrosis in DCM hearts. Unravelling the presence of MH in the absence of coronary stenosis may be helpful to design a novel approach in the clinical management of DCM.

## Introduction

Myocardial hibernation is a well-known feature of ICM. It is clinically characterized by depressed LV resting contractility and perfusion, and recovery of flow-function relations under stress [1, 2]. Even if the presence of viable myocardium has been investigated in end-stage ICM hearts by different methods, the detection of MH structural phenotype represents the essential approach [4]. Such MH phenotype is characterized at cellular and molecular level by glycogen deposition, myocyte cellular hypertrophy, apoptosis, myofibrillar loss and increased expression of ischaemia-responsive proteins [5]–[7]. Although DCM is free from flow-limiting coronary lesions by definition [8], a few consolidated evidence supports a tight association of DCM with endothelial impairment, coronary microvascular dysfunction and reduced flow reserve in response to various stressors [9]–[11]. According to the ‘microvascular ischemic hypothesis’ [9], repeated episodes of ischaemia as a result of perfusion deficit might harm the myocardium and thus play a pathogenic role in the development of DCM [12, 13]. However, it is still controversial whether in DCM, the myocardium might switch to a less vulnerable phenotype resulting in MH in response to a chronic ischaemic deficit. We have previously shown structural hallmarks of MH in tachypacing-induced heart failure (HF) [14], an established animal model of DCM, suggesting that similar alterations might be found in human DCM. So far, no study has clearly shown the presence of direct hallmarks of hibernated myocardium in human DCM, a throughout histological and molecular analysis of explanted hearts being required to investigate such hypothesis [4]. The aim of this study was to fill this lacking information that could provide significant insights into the pathogenesis of DCM; a combined histological and proteomic analysis approach was used to investigate structural and molecular hallmarks of MH in the LV myocardium of end-stage DCM, end-stage ICM and normal hearts (N).

## Materials and methods

### Study population

The study population consisted of 23 patients with HF and end-stage DCM (*n* = 11) or ICM (*n* = 12) eligible for and undergoing heart transplantation (HT) according to recent guidelines [15]. Patients with arterial hypertension, recent myocardial infarction (≤6 months before surgery) or myocarditis, diabetes or evidence of valve disease were excluded from the study. All patients received conventional therapy for end-stage HF and were not chronically treated with high-dose catecholamine infusions. Dilated cardiomyopathy was diagnosed on the basis of echocardiographically documented end-diastolic diameter >56 mm, LV-EF <50% and normal coronary angiography. The selected ICM patients were affected by three-vessel coronary disease. We selected patients with known cardiomegaly of more than 6 months' duration. All patients had waited a similar time on the waiting list for HT. To perform histological and proteomic analysis representative of the LV regions analysed in failing hearts, we used explanted normal hearts (N, *n* = 8, LV-EF >55%), with no history of cardiac disease, selected but not used for transplantation because of unexpected cardiac arrest after brain death. Baseline characteristics of N and HF patients are shown in Table 1. The local ethics review committee approved the study, and the investigation conformed to the Declaration of Helsinki. All patients gave written informed consent before HT to participate in the study.

**Table 1:** Baseline characteristics of normal patients (Control) and HF patients eligible for cardiac transplantation

Variables	Normal (*n* = 11)	DCM (*n* = 11)	ICM (*n* =12)
Age (years)	53 ± 5	41.9 ± 5.45	58.08 ± 1.7
Male	9/11	10/11	12/12
LBBB	0/11	9/11	9/12
PM	0/11	1/11	1/12
Beta-Blockers	–	11/11	8/12
ACE-inhibitors	–	11/11	12/12
Diuretics	–	11/11	12/12
Dobutamine	–	2/11	0/12
Digoxin	–	3/11	4/12
Amiodarone	–	3/11	4/12
NO donor	–	0/11	4/12

DCM, idiopathic dilated cardiomyopathy; ICM, ischaemic cardiomyopathy; LBBB, left bundle branch block; PM, pacemaker; HF, heart failure.

### Coronarographic, haemodynamic and echocardiographic evaluation

All HF patients underwent electrocardiography, coronary angiography and pre-operative non-invasive assessment of coronary arterial pressure, and of cardiac function by transthoracic two-dimensional echocardiography (MY LAB 30; Esaote, Florence, Italy) with a broadband phased array transducer (2–4 MHz). All measurements were performed by an expert cardiologist in accord with the recommendations of the American Society of Echocardiography [16]. We used the ratio between systolic arterial pressure (SAP) and LV end systolic volume (ESV) as a load-insensitive index of systolic global LV function [17]. Systemic vascular resistance (SVR, mm Hg/min./l) was calculated as the ratio of mean arterial pressure to cardiac output [17]. The arterial elastance (Ea, mm Hg/ml) was calculated as: (SAP×0.9)/stroke volume [18].

### Histological and morphometrical analysis

To assess the patency of the epicardial coronary arteries, the hearts were grossly examined as previously described [19]. In all hearts, full-thickness myocardial samples were harvested from the middle third of the IVS and from the left ventricle-free wall (LV-FW), formalin fixed and paraffin embedded. Failing hearts were sampled at the time of HT and normal hearts at the time of donor explantation. For the purposes of this study, areas of gross scarring were avoided in ICM hearts. Five-micrometer serial sections were stained by Haematoxylin-Eosin and Masson's trichrome; Periodic acid-Schiff without (PAS) or with diastase pre-treatment (PAS-D) was used to detect glycogen storage in cardiomyocytes [14]. Morphometric analysis was performed with a computerized image analysis system (OLYMPUS BX 63, Olympus, Milan, Italy) and each parameter (myocyte diameter, collagen deposits, PAS-positivity) was distinctly evaluated in all myocardial (sub-endocardial, mesocardial and subepicardial) layers. In the IVS samples, we separately analysed the sub-endocardial portion belonging to either the right ventricle (RV) and the LV side, and the mesocardial layer. Glycogen storage, assessed as percentage area of PAS-positivity (and absent after diastase treatment), was classified according to the tissue layer localization and to the cellular distribution pattern, *i.e*. cytoplasmic (extending to all cytoplasm on histological section), hob-nail (sub-sarcolemmatic) or granular (mainly peri-nuclear PAS-positive granules). The size of cardiomyocytes was measured in longitudinally sectioned myocytes at 40× magnification in a blinded fashion, as previously described [20, 21]; measurements were performed on the myocytes showing: (*i*) visible and round-shaped nuclei located close to the cell centre; and (*ii*) intact cellular basement membranes. The regional population of normo-, hypo-and hypertrophic cardiomyocytes was expressed as number of cardiomyocytes (10^6^) per gram of cardiac tissue. Masson's trichrome staining was used to detect the interstitial fibrosis, defined as the collagen content determined in the interstitial spaces [22] and to measure the mean percentage area of interstitial fibrosis in 60 fields for specimen at 200× magnification [23].

### TUNEL assay and immunohistochemistry

To detect apoptotic cardiomyocytes, we performed a co-staining of selected LV slides using either TUNEL assay or a mouse monoclonal antibody raised against human alpha-sarcomeric actinin (dilution 1:100; Sigma-Aldrich, Milan, Italy) that is a marker of adult mature cardiomyocytes [24]. We measured the myocardial apoptotic index (number of positive myocytes for field/total myocyte number for field ×100) by TUNEL technique [terminal transferase (TdT) mediated dUTP nick end-labelling] corroborated by the immunohistochemical staining for cleaved caspase-3 on adjacent serial sections [25]. The mean number of capillaries surrounding each cardiomyocyte (number of capillaries/number of cardiomyocytes per field) was determined separately in each LV layer of failing hearts as previously described [16], using anti-Von Willebrand Factor antibody (1:300; Abcam, Cambridge, UK). Connexin 43 (Cx-43) downregulation, a typical feature of MH, was investigated as previously described [26] using a specific polyclonal antibody (dilution 1:1000; Abcam). The foetal intermediate filament nestin, which is re-expressed in human adult cardiomyocytes of infarcted myocardium [27], was revealed using a monoclonal antibody against human nestin (dilution 1:300; Abcam). To detect the nestin-positive cardiomyocytes, we performed a double immunolabelling on selected formalin-fixed LV slices using two specific antibodies raised against human alpha-sarcomeric actinin and human Cx 43 (dilution 1:1000; Abcam), which represents the main connexin isoform in adult cardiomyocytes [28] respectively. To investigate cardiac extracellular matrix turn-over, we also performed specific immunostainings for human collagen type I (by a polyclonal antibody, 1:100 dilution; Santa Cruz Biotechnology, Heidelberg, Germany) [29], human fibronectin, *i.e*., a structural protein of extracellular matrix in hibernated myocardium (dilution 1:200; Millipore, Milan, Italy) [30] and human vimentin (dilution 1:100; Thermo Scientific, Waltham, MA, USA) that has been previously used as a marker of fibroblasts in human hibernating myocardium [31]. Single immunostainings were performed using Avidin-Biotin Complex technique, 3-3′ diaminobenzidine chromogen substrate and Haematoxylin counterstaining, whereas double immunostainings were performed using immunofluorescence technique and DAPI counterstaining.

### Western blotting analysis

Proteins were extracted from snap-frozen myocardial samples of IVS, which represents the most remodelled LV region in the beating failing heart [32]. The membrane was probed with a specific antibody raised against HIF-1α (dilution 1:1000; Santa Cruz Biotechnology), a hallmark of MH in both experimental models and humans [14, 33]. The membranes were reprobed for beta-actin (dilution 1:1000; Santa Cruz Biotechnology) to verify the uniformity of protein loading (see Data S1).

### Proteomic analysis

Human frozen myocardium (5 mg) was collected from viable IVS of ICM (*n* = 5), DCM (*n* = 5) and normal hearts (*n* = 3) and analysed by Multidimensional Protein Identification Technology (MudPIT) approach, as previously described [34, 35] (see Data S1).

### Statistical analysis

All data were analysed using SPSS software (version 13; IBM, Armonk, NY, USA). All quantitative variables are presented as mean ± SEM. One-way anova and Bonferroni post hoc tests were used to compare data between the two groups. A *P* < 0.05 was considered to be statistically significant.

## Results

### Coronarographic, haemodynamic and echocardiographic parameters

As shown in Table 2, no critical stenosis or occlusion was found in the epicardial coronary arteries of DCM patients; in ICM patients, critical stenosis/stenoses were mainly detected in the proximal and middle third of the left anterior descending (LAD) and of the right coronary artery (RCA). Haemodynamic and echocardiographic parameters of global LV function were similar in the DCM and ICM groups (Table 3). Finally, the end-diastolic wall thickness of IVS and LV-FW was similar in HF patients, but significantly reduced as compared with the control group.

**Table 2:** Coronarographic data of HF patients eligible for cardiac transplantation

Variables	DCM (*n* = 11)	ICM (*n* = 12)
Common Trunk of the LCA		
Critical stenosis	–	2/11
Occlusion	–	–
LCX		
Critical stenosis	–	6/11
Occlusion	–	–
Proximal and Middle LAD		
Critical stenosis	–	11/12
Occlusion	–	1/12
Obtuse Marginal Branches		
Critical stenosis	–	5/12
Occlusion	–	4/12
RCA		
Critical Stenosis	–	11/12
Occlusion	–	–

LCA, left coronary artery; LCX, left circumflex artery; LAD, left anterior descending artery; RCA, right coronary artery; HF, heart failure.

**Table 3:** Haemodynamic and echocardiographic parameters of global LV performance

Parameters	Normal (*n* = 11)	DCM (*n* = 11)	ICM (*n* = 12)
HR (bpm)	70 ± 7	78.62 ± 3.9	78.63 ± 2.94
SAP (mmHg)	–	95.9 ± 7.54	97 ± 2.88
DAP (mmHg)	–	65 ± 5.98	61.25 ± 2.5
MAP (mmHg)	–	75.3 ± 5.3	78.3 ± 4.2
DP	–	7540.61 ± 42.76	7624.2 ± 37.48
LVEF (%)	64 ± 7.1	18 ± 3.16*	21.5 ± 6.08*
LVEDV (ml)	100 ± 35	307.5 ± 111.3*	268.5 ± 97.5*
LVESV (ml)	36 ± 12	250 ± 98.8*	217 ± 90.1*
LVSV (ml)	64.3 ± 17	57.5 ± 15.6*	51.5 ± 17.2*
CO (ml/min.)	4501 ± 910	4422.5 ± 1169	4280 ± 1200
SVR (mm Hg/min./l)	–	18.5 ± 4.8	22 ± 10
SAP/LVESV	–	0.42 ± 0.11	0.53 ± 0.15
Ea (mmHg/ml)	–	1.62 ± 0.45	1.9 ± 0.9
LVEDFWT (mm)	11.8 ± 2.1	9.27 ± 1.26*	9.6 ± 2.6*
EDIVST (mm)	11.7 ± 1.3	8.72 ± 1.5*	9.1 ± 3.2*

* *P* < 0.05 *versus* Control.

Data are expressed as mean ± SD.

HR, heart rate; SAP, systolic arterial pressure; DAP, diastolic arterial pressure; MAP, mean arterial pressure; DP, double product; LVEF, left ventricular ejection fraction; LVEDV, left ventricular end-diastolic volume; LVESV, left ventricular end-systolic volume; LVSV, left ventricular stroke volume; CO, cardiac output; SVR, systemic vascular resistance; Ea, arterial elastance; LVEDFWT, left ventricular end-diastolic free wall thickness; EDIVST, left ventricular end-diastolic interventricular septum thickness.

### Left ventricular PAS-positive cardiomyocytes

**Figure 1:**
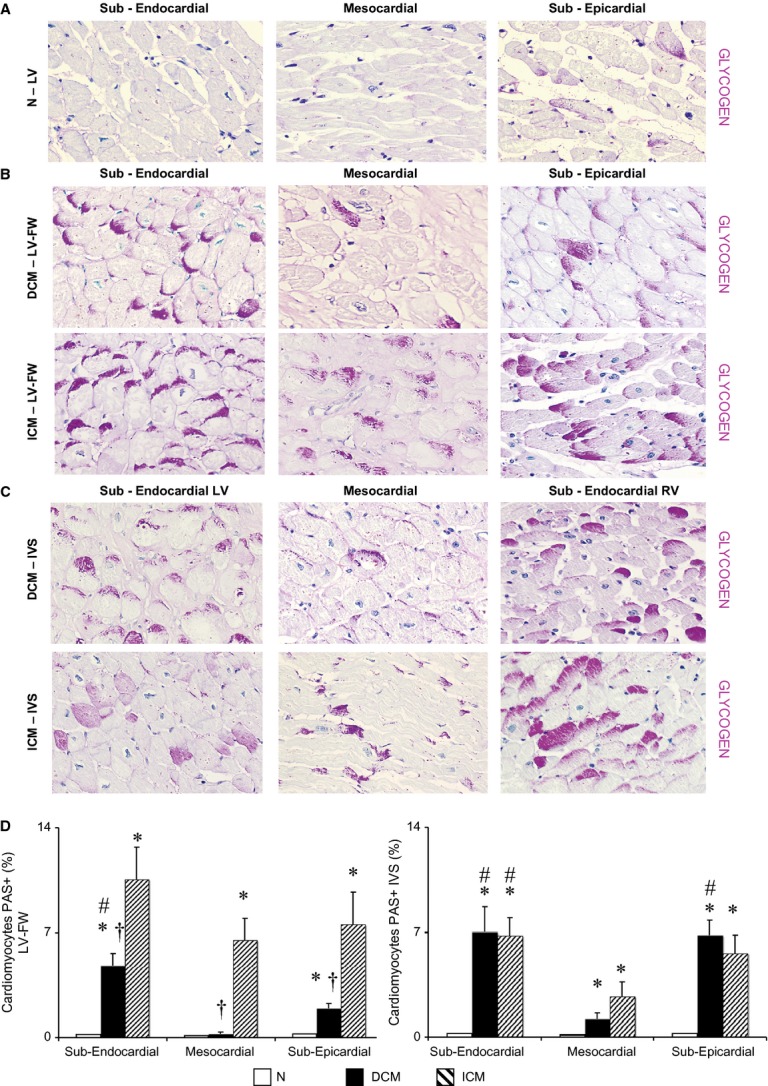
Extent and distribution of regional left ventricle (LV) glycogen deposits as shown by periodic acid-Schiff (PAS) staining on histological sections. (A–C) Representative images of PAS stained sections for each myocardial layer in the LV-free wall (FW) of normal donor (N) hearts, in the LV-FW and in the inter-ventricular septum (IVS) of either dilated cardiomyopathy (DCM) and ischemic cardiomyopathy (ICM) hearts (magnification 400 × ); (D) Intracellular glycogen amount in LV-FW and IVS of N (*n* = 8), of DCM (*n* = 11) and ICM (*n* = 12) hearts. Values are means ± SEM. *P < 0.05 *versus* Normal, ^#^ *versus* mesocardial layer, ^†^*P* < 0.05 *versus* corresponding layer of ICM heart.

Periodic acid-Schiff staining revealed the presence of significant glycogen storage in a larger fraction of myocytes in either the LV-FW or the IVS of both DCM and ICM hearts, and undetectable PAS staining in healthy LV myocardium (data not shown). The zonal distribution of PAS-positivity was similar in the myocardial layers of both failing hearts groups, yet the extent of glycogen deposits in the LV-FW of ICM was higher than in the corresponding layers of DCM hearts (Fig. 1A and B). Conversely, in the IVS, the amount of PAS-positive cardiomyocytes was similar in DCM and ICM hearts (Fig. 1C and D). Moreover, as shown in Figure 1, the population of PAS-positive myocytes was significantly lower in mesocardial layer of DCM hearts. Finally, the intracellular localization of the PAS-positivity showed a variable (cytoplasmic, hob-nail or granular) distribution pattern, independently from HF aetiology (DCM or ICM).

### Left ventricular interstitial fibrosis, collagen type I, fibronectin and vimentin

**Figure 2:**
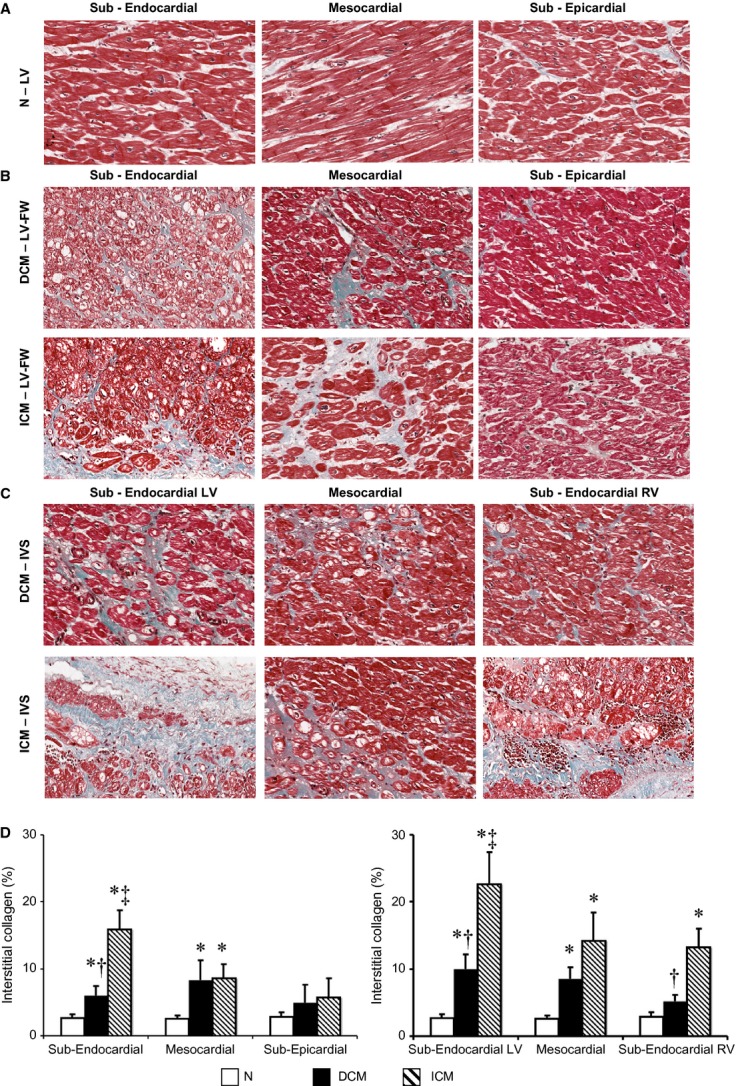
Extent and distribution of regional left ventricle (LV) interstitial collagen. (A–C) Representative images of histological Masson's trichrome-stained sections for each myocardial layer in the LV-free wall (FW) of normal donor (N) hearts, in the LV-FW and in the inter-ventricular septum (IVS) of either dilated cardiomyopathy (DCM) or ischaemic cardiomyopathy (ICM) hearts; (D) Interstitial collagen amount in LV-FW and IVS of N (*n* = 8), of DCM (*n* = 11) and ICM (*n* = 12) hearts. Values are means ± SEM. *P < 0.05 *versus* Normal, ^†^*P* < 0.05 *versus* corresponding layer of ICM heart, ^‡^*P* < 0.05 *versus* sub-epicardial/sub-endocardial right ventricle (RV) layer.

As shown in Figure 2, the interstitial fibrosis was finely distributed in each layer of LV regions in DCM hearts. Conversely, the ICM LV showed higher interstitial fibrosis than DCM hearts. At similar impairment of LV function, fibrosis was significantly increased in sub-endocardial layer of ICM as compared with DCM hearts. In healthy LV myocardium, the interstitial content of collagen was shown to be within the normal range with undetectable myocardial fibrosis (Fig. 2A), according to previous studies [22]. Similarly, higher interstitial amount of type I collagen fibres was detected in the corresponding LV layers of ICM as compared with DCM hearts (Resource S1). Also, the interstitial amount of fibronectin (Resource S2) and fibroblasts (vimentin-positive cells; Resource S3) was finely distributed in all layers of LV regions in DCM hearts, whereas it was larger in ICM LV.

### Left ventricular capillary distribution

**Figure 3:**
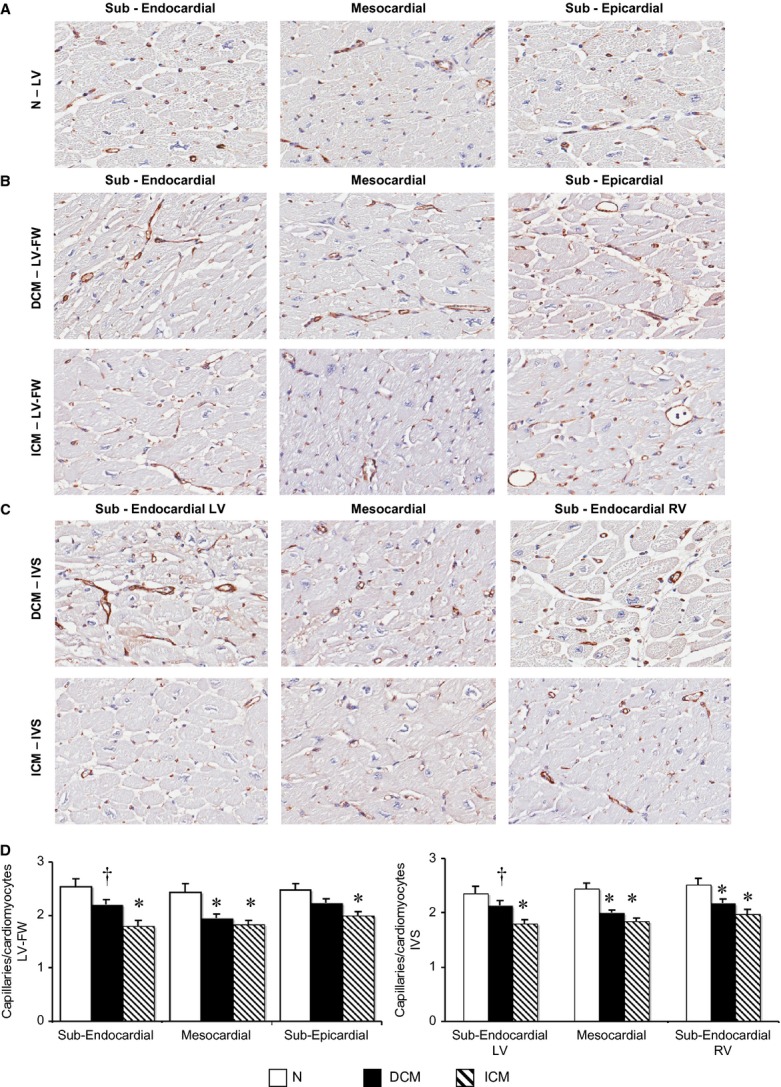
Regional left ventricle (LV) distribution of coronary capillaries surrounding cardiomyocytes. (A–C) Representative images of coronary capillaries detected with an antibody directed against human von Willebrand Factor (vWF) in sections of normal left ventricle (N, *n* = 8) and in LVFW and inter-ventricular septum (IVS) of dilated cardiomyopathy (DCM;*n* = 11) and ischaemic cardiomyopathy (ICM;*n* = 12) hearts; (D) quantification of the number of capillaries per number of cardiomyocytes per field for each myocardial layer. Values are means ± SEM. *P < 0.05 *versus* Normal, ^†^*P* < 0.05 *versus* corresponding layer of ICM heart.

The *ex vivo* coronary examination showed no significant stenosis in the epicardial coronary arteries of either DCM or normal hearts. A homogeneously reduced number of capillaries per cardiomyocyte were detected in all myocardial layers of ICM as compared with N (Fig. 3); in DCM hearts, the LV capillary density was significantly reduced in the mesocardial layer of both IVS and LV-FW, and in the sub-endocardial (RV side) layer of IVS as compared with N heart (Fig. 3D and E). Finally, in DCM, the capillary supply was significantly higher in the sub-endocardial layer of the LV-FW and in the sub-endocardial layer (LV side) of the IVS as compared with the corresponding layers of ICM hearts.

### Left ventricle cardiomyocytes size

**Figure 4:**
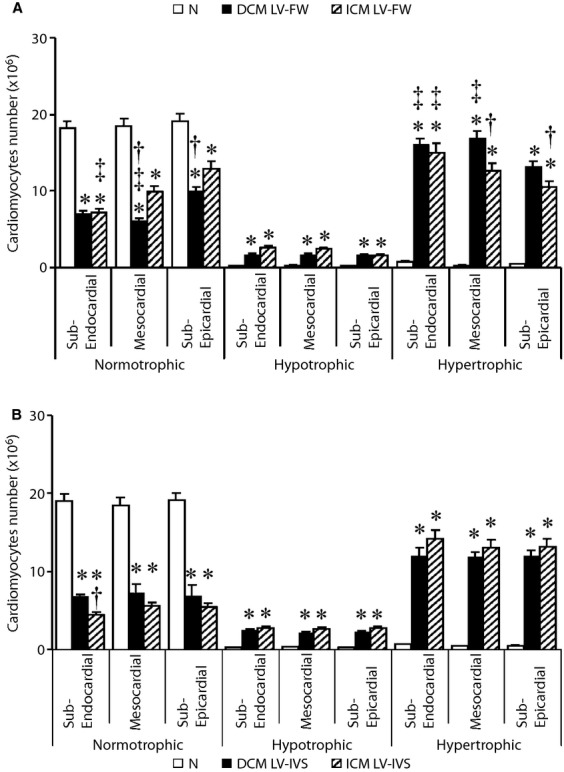
Regional quantification of normo-, hypo-and hyper-trophic cardiomyocytes in each myocardial layer of left ventricle-free wall (LV-FW; A) and inter-ventricular septum (IVS; B) of N (*n* = 8), dilated cardiomyopathy (DCM;*n* = 11) and ischaemic cardiomyopathy (ICM;*n* = 12) hearts. Values are means ± SEM. *P < 0.05 *versus* Normal, ^†^*P* < 0.05 *versus* corresponding layer of ICM heart, ^‡^*P* < 0.05 *versus* sub-epicardial layer/sub-endocardial right ventricle (RV) layer.

As shown in Figure 4, the population of hypertrophic cardiomyocytes was significantly higher than that of normo-and hypo-trophic ones in each LV layer of either DCM or ICM hearts, whereas hypertrophic cardiomyocytes were very rare in N LVs. In addition, the absolute number of hypertrophic cardiomyocytes in mesocardial and sub-epicardial layer of LV-FW in DCM was higher than in ICM hearts (Fig. 4A). Conversely, the population of hypertrophic cardiomyocytes was similarly increased in each layer of LV-IVS of both failing hearts (Fig. 4B). Small population of hypotrophic cardiomyocytes were homogenously detected in each LV layer of both ICM and DCM failing hearts (Fig. 4), yet they were almost undetectable in N LV. Finally, the population of normotrophic myocytes in mesocardial and sub-epicardial layer of LV-FW was more rarefied in DCM than in ICM hearts (Fig. 4A), and the cardiomyocytes with normal size showed a trend to decrease in LV-IVS of ICM as compared with DCM hearts (Fig. 4B).

### Left ventricular expression of connexin 43

**Figure 5:**
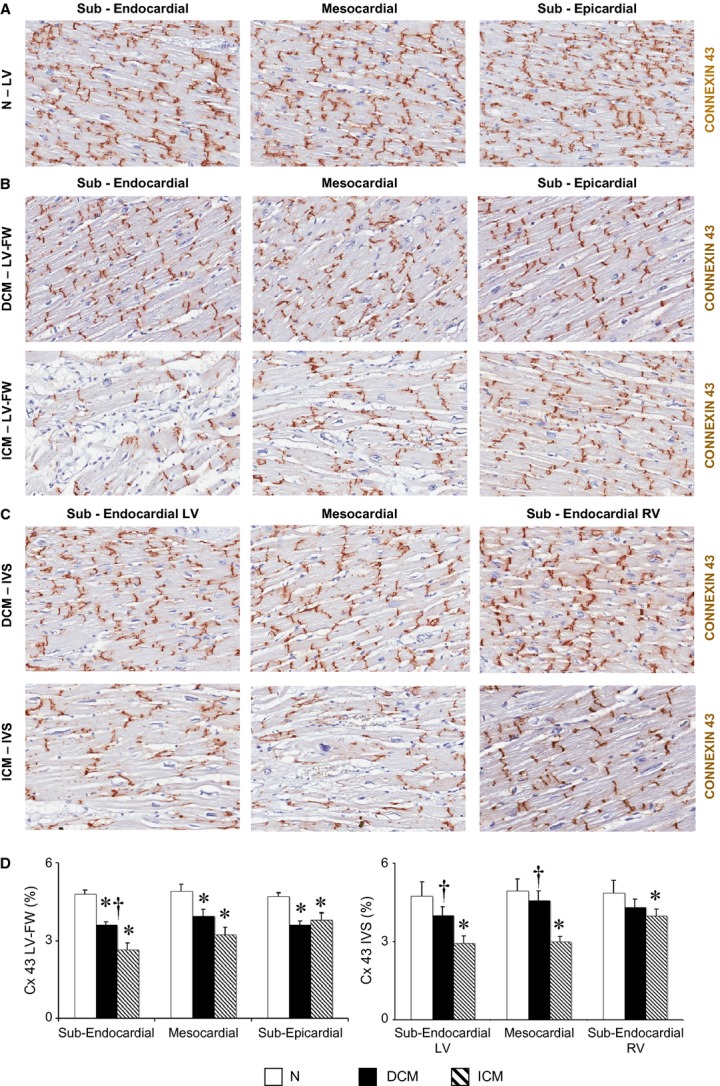
Extent and distribution of regional left ventricle (LV) connexin-43 (Cx 43). (A–C) Representative images of Cx 43 detected by immunohistochemistry in N LV (*n* = 8), and in the LVFW and inter-ventricular septum (IVS) of dilated cardiomyopathy (DCM;*n* = 11) and ischaemic cardiomyopathy (ICM;*n* = 12) hearts; (D) quantification of immunodetectable Cx 43 in each LV myocardial layer. Values are expressed as means ± SEM. *P < 0.05 *versus* Normal, ^†^*P* < 0.05 *versus* corresponding layer of ICM heart.

As shown in Figure 5, the myocardial expression of connexin 43 was homogenously reduced in LV-FW of both failing heart groups as compared with N hearts, yet the distribution of connexin 43 was less detectable in the sub-endocardial layer of ICM as compared with DCM hearts. In addition, the distribution of connexin 43 in IVS of DCM hearts was similar to N hearts, connexin-43 being less detectable in sub-endocardial (LV side) and mesocardial layer of IVS in ICM as compared with DCM.

### Left ventricular apoptotic cardiomyocytes

**Figure 6:**
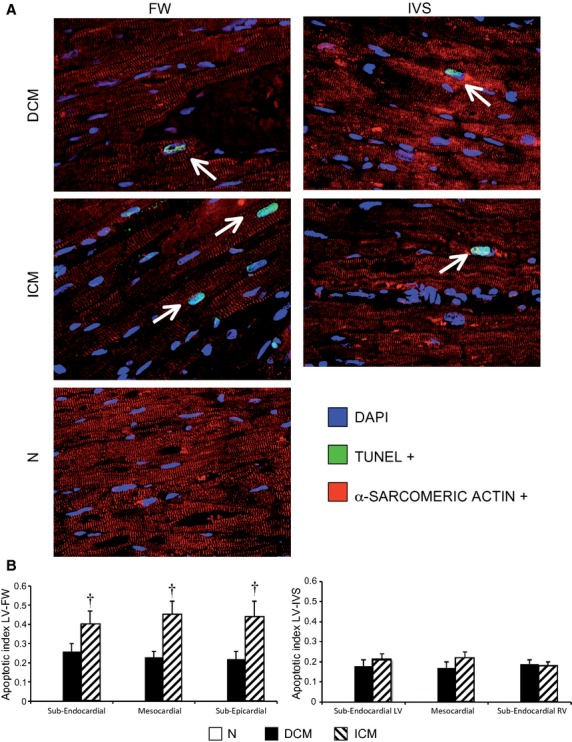
Regional detection of ventricular apoptotic cardiomyocytes (α-SA-positive cells) in left ventricle-free wall (LV-FW) and in inter-ventricular septum (IVS) of dilated cardiomyopathy (DCM;*n* = 11) and ischaemic cardiomyopathy (ICM;*n* = 11) hearts, and N hearts (*n* = 8) by TUNEL assay (A) and regional LV apoptotic index (B). α-SA: alpha-sarcomeric actinin. Values are expressed as means ± SEM. ^†^*P* < 0.05 *versus* corresponding LV layer of DCM heart.

As shown in Figure 6A, we detected apoptotic cardiomyocytes in each LV region of DCM and ICM hearts. The apoptotic index was similar in LV-IVS of the failing hearts, yet it was significantly higher in the LV-FW of ICM as compared with DCM LV-FW (Figure 6B). Apoptotic cardiomyocytes were undetectable in normal hearts, in accord with previous studies [36].

### Lef ventricular expression of hallmarks of ischaemic environment

**Figure 7:**
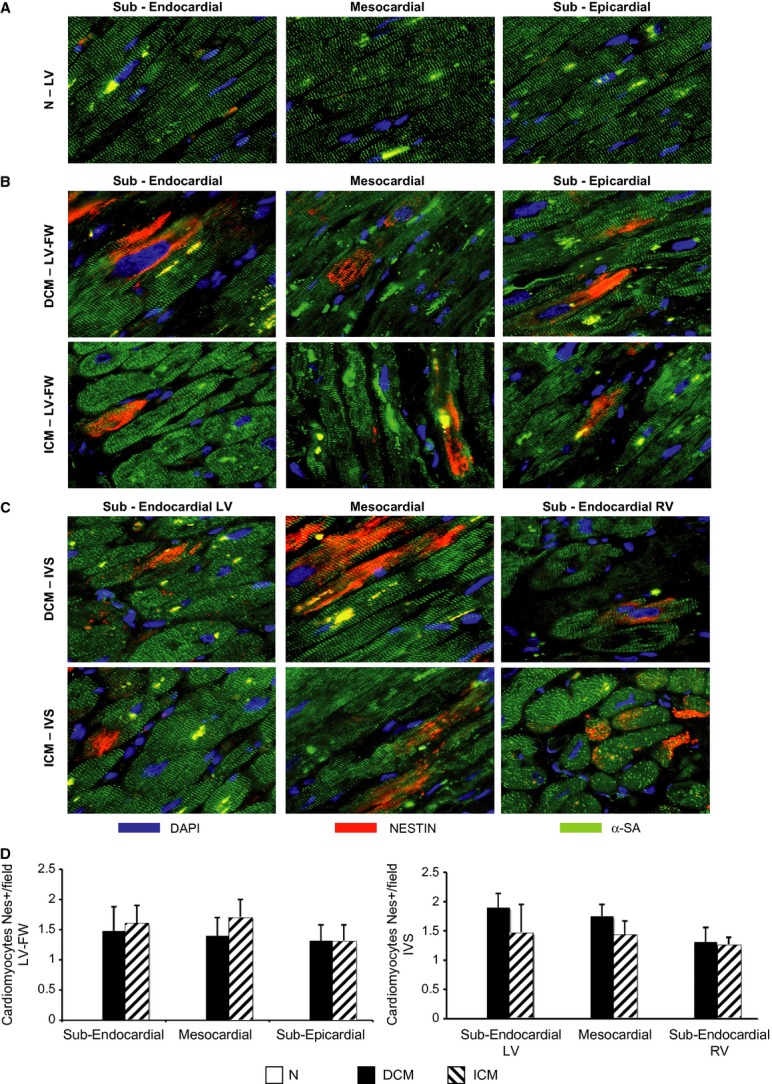
Regional detection of ventricular nestin-positive cardiomyocytes. (A–C) Representative immunofluorescence sections of nestin-positive cardiomyocytes (α-SA-positive cells) in each left ventricle (LV) myocardial layer of N (*n* = 8) and LVFW and inter-ventricular septum (IVS) of dilated cardiomyopathy (DCM;*n* = 11) and ischaemic cardiomyopathy (ICM;*n* = 12) hearts; (D) quantification of nestin-positive cardiomyocytes in each LV myocardial layer of both heart failure groups. α-SA: alpha-sarcomeric actinin. Values are expressed as means ± SEM.

HIF1-α was expressed in both DCM and ICM hearts (0.95 ± 0.16 *versus* 0.82 ± 0.07 a.u.), independently from the magnitude and distribution of myocardial fibrosis. Similarly, Nestin was detected in cardiomyocytes of failing DCM and ICM LV (Fig. 7B and C; Resource S4) and not in normal heart (Fig. 7A; Resource S4).

### Left ventricular proteomic profile

**Figure 8:**
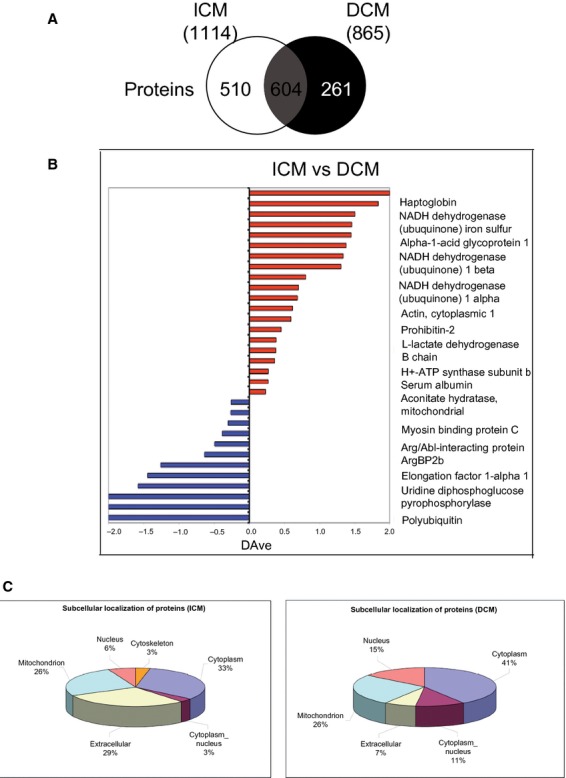
Proteomic profile. (A) Schematic representation of human proteome in inter-ventricular septum (IVS) of both (ischaemic cardiomyopathy, ICM and dilated cardiomyopathy, DCM) failing heart groups showing homology of myocardial proteins. (B) Semiquantitative analysis, in which the bars represent the score ratio expressed by the DAve index. (C) Schematic representation of subcellular distribution of myocardial proteins. *n* = 5 hearts per ICM and DCM group.

The IVS from DCM patients showed protein amounts <22.3 ± 1% as compared to the corresponding region of ICM hearts (Fig. 8). Six hundred proteins expressed into LV-IVS were shared between DCM and ICM hearts. By comparing the protein profiles of failing and normal hearts, 12 proteins were found to be up-regulated in DCM-IVS and 20 proteins in ICM-IVS (Table 4), the up-regulated proteins of ICM being down regulated in DCM, and *vice versa* (Fig. 8B). The up-regulated proteins in ICM were down-regulated in DCM, and *vice versa* (Fig. 8B). In particular, uridine diphospho-glucose pyrophosphorylase, a glycogen synthesizing enzyme [37], alpha-crystallin B, which is a cardioprotective small heat shock protein [38], aconitate hydratase, a mitochondrial enzyme of citric acid cycle that is increased in cardiomyocytes exposed to intermittent hypoxia [39] and to increased pre-load [40], were significantly up-regulated in the DCM myocardium as compared with ICM hearts. By analysing the distribution of subcellular and extracellular myocardial proteins, the amount of extracellular proteins was found to be higher in ICM than in DCM myocardium (see Fig. 8C).

**Table 4:** Proteomic profile of LV-IVS of DCM and ICM failing hearts

Accession	Reference	pI	MW	*t*-test (*P* > 95%)	ISC (SpC)	DIL (SpC)	*G*-test (*P* > 95%)	DAVE	DCI		Uniprot
881394	Uridine diphosphoglucose pyrophosphorylase	8.3	55702	*	0.00	0.59		−2.00	−17	Up-regulated in Dilated	Q16851
2627129	Polyubiquitin	7.7	68492	*	0.00	1.09		−2.00	−59		P0CG48
7023143	2-Oxoglutarate dehydrogenase-like	6.2	114480	*	0.00	0.81		−2.00	−33		Q9ULD0
10584324	Xin actin-binding repeat-containing protein 1	6.8	112075	*	0.11	0.97		−1.58	−46		Q702N8
4503471	Elongation factor 1-alpha 1	9.4	50142	*	0.22	1.36		−1.45	−90		P68104
4504183	Glutathione *S*-transferase P	5.3	23357	*	0.46	2.04		−1.26	−197		P09211
2952333	Arg/Abl-interacting protein ArgBP2b	9.9	70661	*	2.23	4.32		−0.64	−682		O94875
4503057	Alpha-crystallin B chain	6.9	20160		50.74	83.98	*	−0.49	−223887		P02511
2058322	Myosin binding protein C gene	6.2	140748		41.55	61.38	*	−0.39	−102089		Q14896
115496169	Myosin-7	5.5	223097		100.36	135.90	*	−0.30	−419833		P12883
4501867	Aconitate hydratase, mitochondrial	7.3	85407	*	56.36	73.39		−0.26	−110483		Q99798
297024	Cardiac alpha-myosin heavy chain	5.5	223705		257.64	334.43	*	−0.26	−2273187		P13533
4502027	Serum albumin	5.9	69367		248.82	197.58	*	0.23	1143714	Up-regulated in Ischemic	P02768
14016	Cytochrome *c* oxidase II subunit	4.5	20995	*	15.27	11.65		0.27	4878		P00403
509291	H^+^-ATP synthase subunit b	9.7	28895	*	18.83	14.33		0.27	7455		P24539
4503607	Electron transfer flavoprotein subunit alpha	8.5	35061	*	28.55	19.81		0.36	21126		P13804
4557032	l-lactate dehydrogenase B chain	5.7	36639	*	56.29	38.42		0.38	84607		P07195
190885499	Cytochrome *c* oxidase subunit 5A	6.4	16744	*	31.38	21.32		0.38	26506		P20674
6005854	Prohibitin-2	10.2	33297	*	8.77	5.53		0.45	2316		Q99623
46593007	Cytochrome *b*–*c*1 complex	5.9	52627	*	9.58	5.22		0.59	3227		P31930
4501885	Actin, cytoplasmic 1	5.2	41738	*	9.41	4.99		0.61	3183		P60709
4503143	Cathepsin D	6.1	44553	*	12.07	5.92		0.68	5532		P07339
6681764	NADH dehydrogenase [ubiquinone] 1 alpha	10.2	42491	*	6.64	3.20		0.70	1689		Q16795
50363217	Alpha-1-antitrypsin	5.3	46737		19.94	8.55	*	0.80	16216		P01009
4758774	NADH dehydrogenase [ubiquinone] 1 beta	8.6	20759	*	1.49	0.31		1.30	106		O96000
2414492	Immunoglobulin heavy chain, constant region	6.0	25732	*	3.69	0.74		1.33	654		
167857790	Alpha-1-acid glycoprotein 1	4.9	23521	*	4.07	0.75		1.38	799		P02768
7705501	Transmembrane protein 14C	10.2	11566	*	0.72	0.12		1.45	25		Q9P0S9
4758786	NADH dehydrogenase [ubiquinone] iron-sulfur	7.3	52527	*	0.49	0.08		1.45	12		O75306
40789020	KIAA1009	5.4	155046	*	1.63	0.23		1.50	130		Q5TB80
1620396	Haptoglobin	6.4	39008	*	1.89	0.08		1.84	179		P00739
223029410	Talin-1	5.7	269766	*	0.56	0.00		2.00	16		Q9Y490

Specific myocardial proteins were identified by comparing the protein lists of DCM-and ICM-IVS against the average protein profile of normal cardiac tissue, by means of MAProMa software (through calculation of Dave/DCI values). The up-regulated proteins in DCM were down-regulated in ICM, and vice versa. pI, isolectric point; MW, molecular weight.

* *P*-value statistically significant.

## Discussion

In this study, the structural hallmarks of chronic MH were detected in the LV FW and in the IVS of end-stage DCM hearts with coronary patency and left bundle branch block (LBBB); they were also shown to be associated with molecular features of ischaemic microenvironment. Similar hallmarks of chronic MH were also detectable in the corresponding LV regions of end-stage ICM hearts harvested from patients with flow-limiting, critically stenotic and diffuse lesions of the epicardial coronary arteries, and LBBB. The phenotype of chronic MH was more pronounced in ICM hearts that constituted the positive control group of this study. The lack of ischaemic microenvironment and MH phenotype in the explanted normal hearts allowed the exclusion of an ischaemic damage depending on the brain death time, surgical procedure or the cardiac allograft storage. These findings support the hypothesis that the long-term exposure of adult human myocardium to an ischaemic microenvironment triggers biochemical processes leading up to hibernation, by interfering on gene and protein myocardial expression and causing the typical histological alterations that altogether represent a compensatory mechanism aimed at maintaining myocardial viability despite reduced myocardial blood flow [2].

The development of the chronic MH phenotype in HF patients is difficult to track, whereas the morphological features of chronic MH are well-established and characterized by PAS-positive cardiomyocytes, loss of cardiomyocytes by apoptosis, patchy reduction in Cx-43 content (contributing to impaired myocardial excitation-contraction coupling), hypertrophic and hypo-/atrophic cardiomyocytes, interstitial fibrosis, increased fibronectin and fibroblasts (vimentin-positive cells) [2].

Although imaging-based diagnostic tests identified viable and dysfunctional myocardium in LV regions of patients with end-stage DCM [41, 42], the *ex vivo* regional characterization of chronic MH phenotype was never performed in full-thickness myocardial samples from human failing DCM hearts so far. This study is the first to show the patchy structural hallmarks of chronic MH in the myocardial layers of full-thickness myocardial samples collected from two different LV regions of failing human DCM hearts that are characterized by patent epicardial coronary arteries.

The myocardial extent of intracellular glycogen, a clinically relevant marker of MH detected by PAS staining [6, 14], showed a U-shaped distribution with larger amounts in the ICM LV-FW as compared with DCM LV-FW, associated with higher content of apoptotic cardiomyocytes and of interstitial fibrosis. Conversely, the myocardial glycogen amount and the number of apoptotic cardiomyocytes were similar in the IVS of both failing heart groups (ICM and DCM) despite lower interstitial fibrosis in DCM as compared with ICM hearts. Even if it is very difficult to determine the natural history of MH in HF patients [2, 43], it is conceivable that the chronic MH phenotype in HF patients was primed and maintained by repeated episodes of ischaemia and subsequent cumulative stunning [7]. The magnitude of the hibernating phenotype was enhanced in ICM patients that have occlusions of epicardial coronary arteries [31], whereas the smaller extent of the chronic MH phenotype in DCM failing hearts was very likely related to the selective dysfunction of coronary microcirculation [44]. Accordingly, we found larger increase in type I collagen, fibronectin and fibroblasts in ICM rather than in DCM hearts. The patchy expression of chronic MH phenotype in DCM hearts might correspond to a mirroring distribution of the ischaemic microenvironment depending on the impairment of the coronary microcirculation rather than to the main coronary arteries perfusion areas. In our DCM failing hearts, the presence of an ischaemic microenvironment was proved by the myocardial expression of HIF-1alpha and nestin. As HIF-1 alpha is a transcription factor that mediates cardiac adaptive responses to hypoxia/ischaemia [43] and represents an established molecular hallmark of chronic MH in failing heart of animal model and of patients as well [14, 33], it is noteworthy that in this study, HIF-1 alpha was similarly expressed in both DCM and ICM hearts.

The main adaptive response to hypoxia/ischaemia observed in adult myocardium chronically exposed to ischaemic microenvironment is the re-expression of foetal genes that has been previously shown in failing adult LV as a result of chronic MH [45, 46].

Nestin is a foetal intermediate filament and an endogenous inhibitor of apoptosis [47], which is re-expressed in adult human ventricular myocytes exposed to chronic ischaemia [27]. We detected an equal number of nestin-positive cardiomyocytes in both DCM and ICM hearts, these data supporting the hypothesis that a foetal gene pattern is expressed in chronically hibernating heart.

A high number of hypertrophic cardiomyocytes were found in the LV viable myocardium of DCM hearts. Hypertrophic cardiomyocytes are considered a hallmark of cellular remodelling in response to apoptosis [36] and/or to ischaemic signalling [48]. Recent evidence has shown that ischaemic paracrine mediators lead to cellular hypertrophy and to reduced expression of connexin43 in cardiomyocytes bordering the ischaemic area [48], both changes underlying the electro-mechanical decoupling typical of the hibernating myocardium [49]. In particular, the depletion of Cx-43 plays a pathogenic role in the chronic MH [26]. In our DCM patients, we detected patchy depletion of myocardial connexin-43 in the LV layers; this finding well represents the jeopardized expression of the structural hallmarks in chronic MHe.

On the basis of the previous findings, we suggest that the chronic maintenance of the MH phenotype was strictly dependent on the presence of an ischaemic microenvironment even in DCM hearts.

Even though the myocardial adaptive response to ischaemic microenvironment may also be independent of haemodynamic factors as a result of temporal changes of LV function [11, 50], the abovementioned structural phenotype in DCM failing hearts might depend on the impairment of coronary microcirculation. The LV capillary density was similarly reduced at the mesocardial level in both DCM and ICM failing hearts; however, the myocardial microcirculation was less impaired in the LV sub-endocardial layer of DCM compared with ICM hearts. Interestingly, the mesocardial capillary supply of DCM hearts was significantly reduced compared with N in the presence of minimal interstitial fibrosis and lower intracellular amount of glycogen.

Despite established evidence of severe myocardial hypoperfusion [11, 13, 41] and defective vessel formation [51] in the LV of patients with end-stage DCM, the local capillary/glycogen mismatch observed by us supports the hypothesis that the exposure of myocardium to persistent ischaemic environment was because of dysfunction rather than to reduction in coronary capillary network in LV regions of end-stage DCM heart [52].

To elucidate the coronary microvascular response regardless of the presence of coronary lesions or capillary depletion in DCM hearts, we can suggest that chronic LV dyssynchrony following Cx-43 depletion, typical of patients with LBBB and severe contractile dysfunction [53], might affect the myocardial viability [54].

Despite a recent imaging study did not show consistent changes of myocardial blood flow in dyssynchronous failing heart of DCM patients [55], we previously detected functional, histological and molecular hallmarks of MH in the less perfused and dyssynchronous LV region of a swine model of non-ischaemic HF [14], where prolonged LV dyssynchrony leads to myocardial hypoperfusion [56].

Similar to the abovementioned hallmarks of MH, the regional loss of Cx-43 was also smaller in the end-stage DCM compared with ICM hearts.

To better define the chronic MH phenotype in human end-stage DCM, we performed a detailed proteomic characterization of full-thickness samples collected from IVS, which is the LV region mainly affected by structural remodelling in the dyssynchronous failing heart [32] and by broader loss of Cx-43 (Fig. 5). Interestingly, the amount of functionally and structurally intact proteins was reduced by 20% in DCM as compared with ICM hearts. It is conceivable that an increased myocardial activation of proteases takes place in DCM, promoting cellular remodelling and cardiac dysfunction because of loss of key proteins [57], such as Cx-43. Finally, we found a 70% homology between intact proteins of DCM and ICM hearts. It is remarkable that alpha-crystallin B [38] and aconitate hydratase [39], which are overexpressed in response to ischaemic microenvironment, and uridin diphospho-glucose pyrophosphorylase, a glycogen synthesizing enzyme [37], were up-regulated in the presence of reduced capillary density, minimal interstitial fibrosis and Cx-43 depletion in IVS-DCM. Therefore, we cannot exclude that the progressive rearrangement or disruption of cell junctions enables the activation of ischaemic signalling leading to chronic MH towards degradation of cellular pathways.

### Limits of our study

Our study did not provide direct information as to the relationship between histological and functional features of explanted hearts. Finally, the limited sample size of patients without LBBB precluded a comparative analysis between DCM patients with and without LBBB. Then, further investigations are mandatory to better define if the abovementioned histological and morphometric changes might be related to the chronic nature of the HF more than to the mechanisms of chronic MH.

### Conclusions

Structural features of chronic MH that is typical of ischaemic chronic HF were identified in the LV of patients with end-stage DCM undergoing HT, associated with evidence of ischaemic micro-environment and dyssynchronous substrate. Our investigation provides new data by a multimodal *ex vivo* analysis.

In conclusion, unravelling the basic molecular mechanisms leading to MH in DCM hearts may be helpful to identify novel pharmacological targets to halt the progression of remodelling towards HF, especially in patients not responsive to conventional treatments.

## Supporting information

**Resource S1.** Extent and distribution of regional LV interstitial type I collagen. a, b, c: representative images of collagen type I detected with immunohistochemical staining of sections of N (n = 8) and LVFW and IVS of DCM (n = 11) and ICM (n = 12) hearts; d: quantification of immunodetectable collagen type I in each LV myocardial layer. Values are means ± SEM. *P > 0.05 versus Normal, ^†^P > 0.05 versus corresponding layer of ICM heart, ^‡^P > 0.05 versus sub-epicardial layer/sub-endocardial RV layer.

**Resource S2.** Extent and distribution of regional LV interstitial fibronectin. a, b, c: representative images of fibronectin detected with immunohistochemical staining of sections of N (n = 8) and LVFW and IVS of DCM (n = 11) and ICM (n = 12) hearts; d: quantification of immunodetectable fibronectin in each LV myocardial layer. Values are means ± SEM. *P > 0.05 versus Normal.

**Resource S3.** Extent and distribution of regional LV interstitial vimentin-positive cells. a, b, c: representative images of vimentin-positive cells detected with immunohistochemical staining of sections of N (n = 8) and LVFW and IVS of DCM (n = 11) and ICM (n = 12) hearts; d: quantification of immunodetectable vimentin-positive cells in each LV myocardial layer. Values are means ± SEM. *P > 0.05 versus Normal.

**Resource S4.** Regional detection of ventricular nestin-positive cardiomyocytes. a,b,c: representative immunofluorescence sections of nestin-positive cardiomyocytes (cx-43-positive cells) in each LV myocardial layer of N (n = 8) and LVFW and IVS of DCM (n = 11) and ICM (n = 12) hearts. Cx-43: connexin-43.

**Data S1.** Expanded methods.
