# Supplementary figures and images for: Regional mapping of myocardial hibernation phenotype in idiopathic end-stage dilated cardiomyopathy
Source: J Cell Mol Med. 2014 Jan 20;18(3):396–414. doi: 10.1111/jcmm.12198 (PMC3955147; doi:10.1111/jcmm.12198)

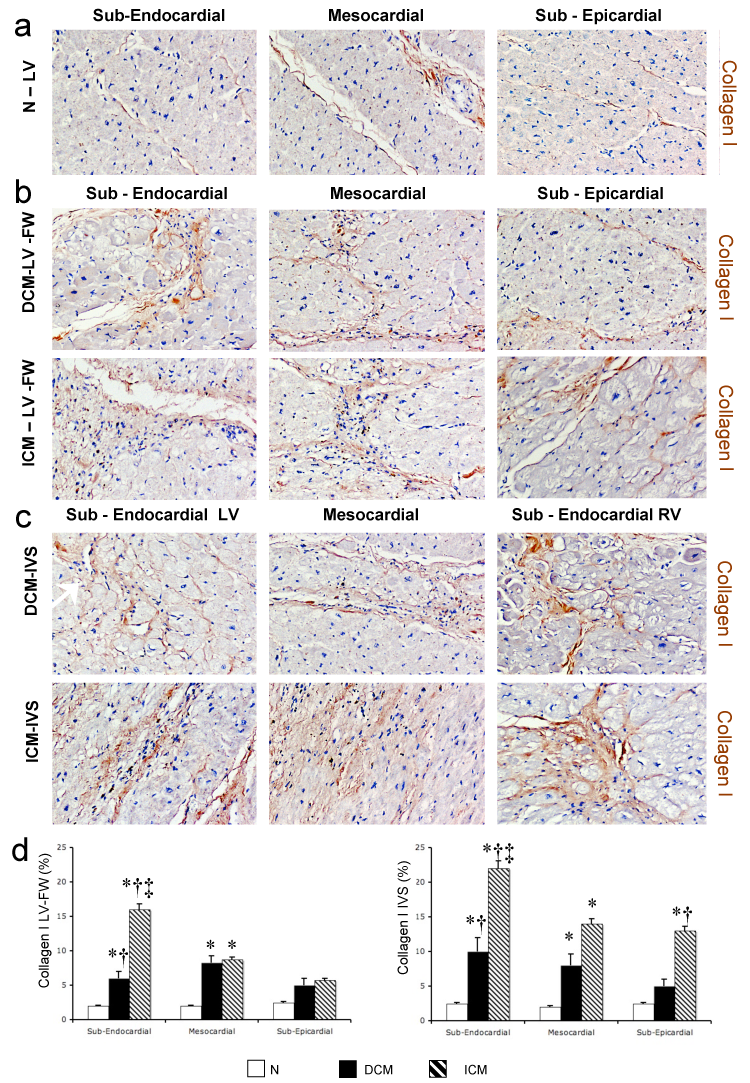

Supplement: Supplementary file 1 — Resource S1. Extent and distribution of regional LV interstitial type I collagen. a, b, c: representative images of collagen type I detected with immunohistochemical staining of sections of N (n = 8) and LVFW and IVS of DCM (n = 11) and ICM (n = 12) hearts; d: quantification of immunodetectable collagen type I in each LV myocardial layer. Values are means ± SEM. *P > 0.05 versus Normal, †P > 0.05 versus corresponding layer of ICM heart, ‡P > 0.05 versus sub-epicardial layer/sub-endocardial RV layer. [file jcmm0018-0396-sd1.tif]

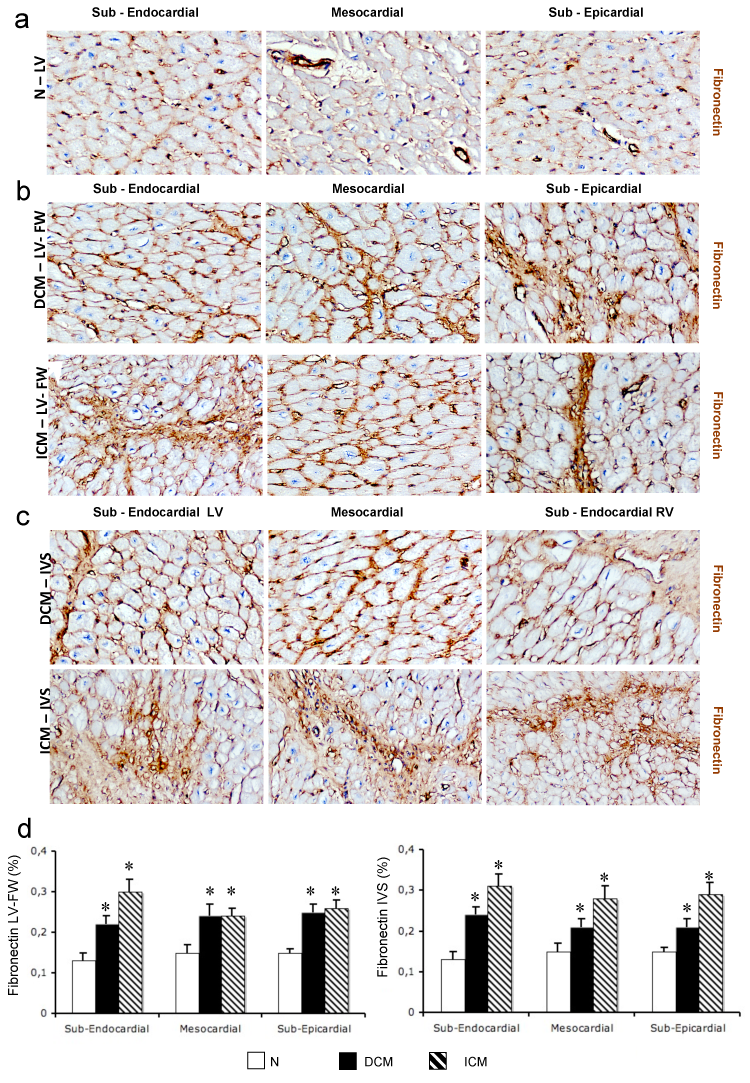

Supplement: Supplementary file 2 — Resource S2. Extent and distribution of regional LV interstitial fibronectin. a, b, c: representative images of fibronectin detected with immunohistochemical staining of sections of N (n = 8) and LVFW and IVS of DCM (n = 11) and ICM (n = 12) hearts; d: quantification of immunodetectable fibronectin in each LV myocardial layer. Values are means ± SEM. *P > 0.05 versus Normal. [file jcmm0018-0396-sd2.tif]

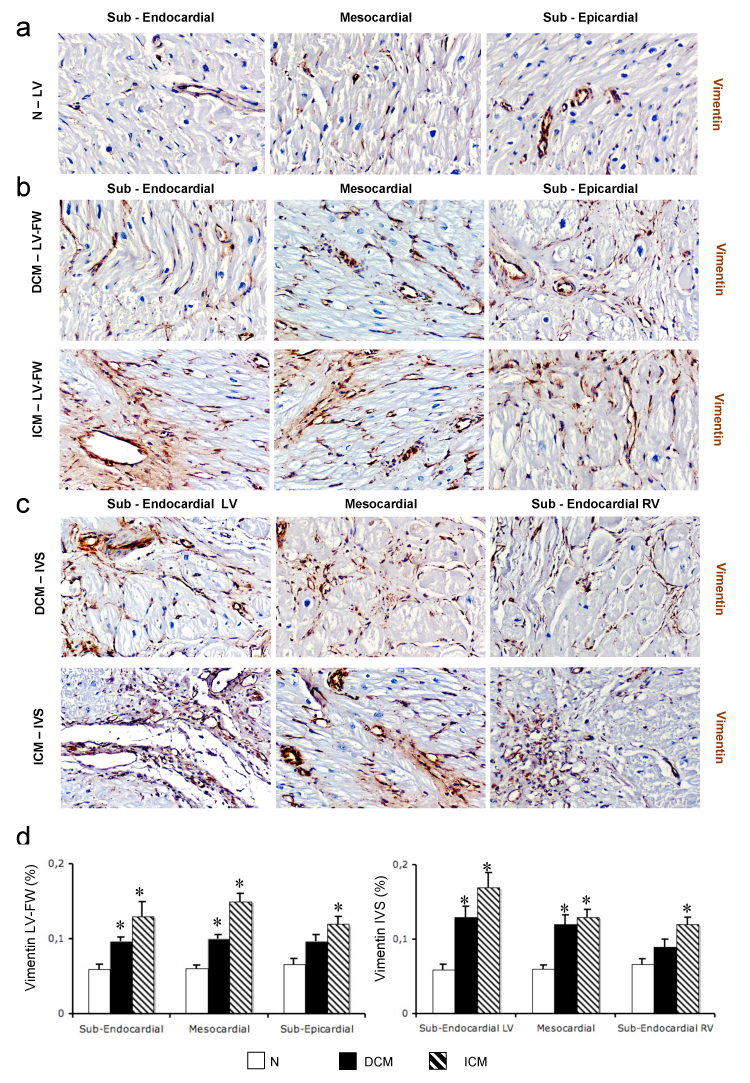

Supplement: Supplementary file 3 — Resource S3. Extent and distribution of regional LV interstitial vimentin-positive cells. a, b, c: representative images of vimentin-positive cells detected with immunohistochemical staining of sections of N (n = 8) and LVFW and IVS of DCM (n = 11) and ICM (n = 12) hearts; d: quantification of immunodetectable vimentin-positive cells in each LV myocardial layer. Values are means ± SEM. *P > 0.05 versus Normal. [file jcmm0018-0396-sd3.tif]

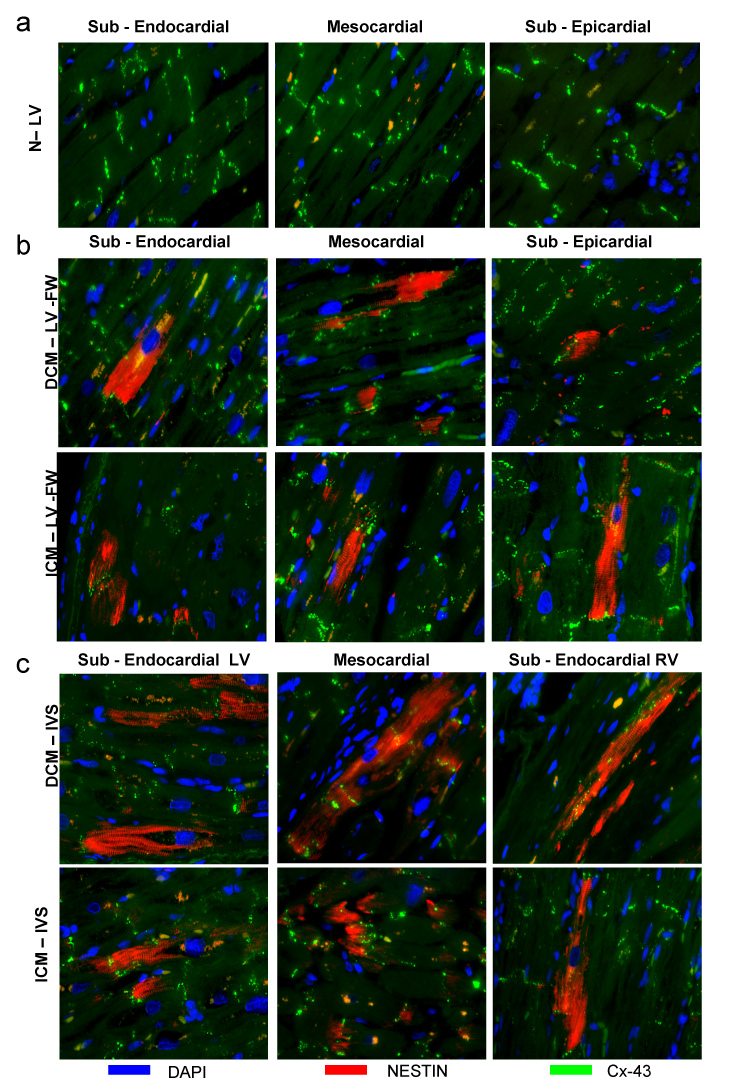

Supplement: Supplementary file 4 — Resource S4. Regional detection of ventricular nestin-positive cardiomyocytes. a,b,c: representative immunofluorescence sections of nestin-positive cardiomyocytes (cx-43-positive cells) in each LV myocardial layer of N (n = 8) and LVFW and IVS of DCM (n = 11) and ICM (n = 12) hearts. Cx-43: connexin-43. [file jcmm0018-0396-sd4.tif]
