# Supplementary material for: Regional mapping of myocardial hibernation phenotype in idiopathic end-stage dilated cardiomyopathy
Source: J Cell Mol Med. 2014 Jan 20;18(3):396–414. doi: 10.1111/jcmm.12198 (PMC3955147; doi:10.1111/jcmm.12198)
Supplement: Supplementary file 5 — Data S1. Expanded methods. [file jcmm0018-0396-sd5.docx]

ONLINE DATA SUPPLEMENT

**EXPANDED METHODS**

*Western blotting analysis*

Fifty microgram of total protein was resolved by sodium dodecyl sulfate polyacrylamide gel electrophoresis on 10% gel and blotted electrophoretically. After probing with the secondary peroxidase-conjugated antibody, the protein bands were developed in a chemiluminescence substrate solution (Pierce SuperSignal Chemiluminescents Substrate).

*Proteomyc analysis*

The mass spectra were compared to *human* peptide sequences downloaded from NCBI website ([www.ncbi.nlm.nih.gov](http://www.ncbi.nlm.nih.gov/)). The first best-matching peptide was taken into consideration in accord to recent guidelines [1].

The proteomic profiles, first identified by Sequest Score, were automatically clustered by Ward method distance and analyzed using MAProMA (Multidimensional Algorithm Protein Map) software [3]. The DAve (Differential average) algorithm, which evaluates the changes in protein expression, and DCI (Differential Confidence index), which describe the confidence of differential expression, were used for a semi-quantitative evaluation of relative differential expression of identified peptides [3]. The confidence of protein identification was based on the high stringency applied for the identification and matching of the peptide sequence, as previously described [2]. Moreover, identified proteins were listed and plotted on the basis of molecular weights (MW) and isoelectric point (pI). In the present study, we performed the Total Signal normalization of proteomic data on the base of Spectral Count (SpC) and Sequest Score in order to reduce false positive/negative identification of differentially represented proteins. We employed statistical G-test (p<0.05) to analyse spectral count values to better support the used criteria of selection, as previously described [2].

**REFERENCE**

1. Briani F, Curti S, Rossi F, Carzaniga T, Mauri P, Dehò G. Polynucleotide phosphorylase hinders mRNA degradation upon ribosomal protein S1 overexpression in Escherichia coli. RNA 2008; 14:2417-2429.
2. Simioniuc A, Campan M, Lionetti V et al. Placental stem cells pre-treated with a hyaluronan mixed ester of butyric and retinoic acid to cure infarcted pig hearts: a multimodal study. Cardiovasc Res 2011; 90:546-556.
3. Zhang B, VerBerkmoes NC, Langston MA, Uberbacher E, Hettich RL, Samatova NF. Detecting Differential and Correlated Protein Expression in Label-Free Shotgun Proteomics. J Proteome Res 2006; 5: 2909-2918.

**ONLINE RESOURCE LEGENDS**

**Online Resource 1.** Extent and distribution of regional LV interstitial type I collagen. **a, b, c**: representative images of collagen type I detected with immunohistochemical staining of sections of N (n=8) and LVFW and IVS of DCM (n=11) and ICM (n=12) hearts; **d:** quantification of immunodetectable collagen type I in each LV myocardial layer. Values are means ± S.E.M. * P<0.05 vs. Normal, ^†^ P<0.05 vs. corresponding layer of ICM heart, ‡ P<0.05 vs. sub-epicardial layer/sub-endocardial RV layer.

**Online Resource 2.** Extent and distribution of regional LV interstitial fibronectin. **a, b, c**: representative images of fibronectin detected with immunohistochemical staining of sections of N (n=8) and LVFW and IVS of DCM (n=11) and ICM (n=12) hearts; **d:** quantification of immunodetectable fibronectin in each LV myocardial layer. Values are means ± S.E.M. * P<0.05 vs. Normal.

**Online Resource 3.** Extent and distribution of regional LV interstitial vimentin-positive cells. **a, b, c**: representative images of vimentin-positive cells detected with immunohistochemical staining of sections of N (n=8) and LVFW and IVS of DCM (n=11) and ICM (n=12) hearts; **d:** quantification of immunodetectable vimentin-positive cells in each LV myocardial layer. Values are means ± S.E.M. * P<0.05 vs. Normal.

**Online Resource 4.** Regional detection of ventricular nestin-positive cardiomyocytes. **a,b,c**: representative immunofluorescence sections of nestin-positive cardiomyocytes (cx-43-positive cells) in each LV myocardial layer of N (n=8) and LVFW and IVS of DCM (n=11) and ICM (n=12) hearts. Cx-43: connexin-43.
